# Pre-clinical testing of malignant fibroblast activation protein-specific re-directed T cells for treatment of pleural mesothelioma

**DOI:** 10.1186/2051-1426-1-S1-P35

**Published:** 2013-11-07

**Authors:** Petra C Schuberth, Christian Hagedorn, Shawn M Jensen, Pratiksha Gulati, Osiris Marroquin Belaunzaran, Alex Soltermann, Astrid Jüngel, Christoph Renner, Ulf Petrausch

**Affiliations:** 1 Department of Oncology, University Hospital Zurich, Zurich, Switzerland; 2 Laboratory of Molecular and Tumor Immunology, Earle A. Chiles Research Institute, Portland, OR, USA; 3 Institute of Surgical Pathology, University Hospital Zurich, Zurich, Switzerland; 4 Center of Experimental Rheumatology, University Hospital Zurich, Zurich, Switzerland; 5 Department of Oncology, University Hospital Basel, Basel, Switzerland; 6 Department of Immunology, University Hospital Zurich, Zurich, Switzerland

## Introduction

Malignant pleural mesothelioma (MPM) is an incurable malignant disease that results mostly from chronic exposition to asbestos. Fibroblast activation protein (FAP) is predominantly expressed on the surface of reactive tumor-associated fibroblasts and on particular cancer types. Therefore, FAP is an attractive target for adoptive T cell therapy. T cells can be re-directed by gene transfer of FAP-specific chimeric antigen receptors (CAR).

## Methods

Immunohistochemistry was performed on tumor tissue from MPM patients to evaluate FAP expression. CD8+ human T cells were retrovirally transduced with an anti-FAP-F19-ΔCD28/CD3ζ-CAR constructs. T cell function was evaluated by cytokine release and cytotoxicity assays *in vitro*. *In vivo* function was tested with an intraperitoneal xenograft tumor model.

## Results

FAP was found to be expressed in all subtypes of MPM. FAP expression was evaluated in healthy adult tissue samples and detected in pancreas and placenta. FAP-specific re-directed T cells lysed FAP positive mesothelioma cells and inflammatory fibroblasts in an antigen-specific manner *in vitro*. FAP-specific re-directed T cells inhibited the growth of FAP positive human tumor cells in the peritoneal cavity of mice and significantly prolonged survival of mice. We plan to adoptively transfer re-directed T cells into the pleural cavity of MPM patients treated with chemotherapy. Cytokine analysis of pleural effusions from patients revealed presence of TGF-β1, VEGF, IL-6, and IL-10. Incubation of re-directed T cells with pleural effusions resulted in suppressed antigen-specific IFNγ secretion in 2 of 9 cases. FAP specific re-directed T cells were incubated with chemotherapeutic agents at reported plasma peak concentrations. No decrease in IFNγ secretion was detected for pemetrexed. Higher concentrations of cisplatin reduced IFNγ secretion by re-directed T cells.

## Conclusion

FAP re-directed CD8+ T cells showed antigen-specific functionality *in vitro* and *in vivo*. Furthermore, FAP expression was verified in all MPM subtypes and IFNγ secretion of re-directed T cells was not hampered in most cases if incubated with pleural effusions from patients. Therefore, our data support the conduct of a phase I clinical trial with adoptively transferred FAP-specific re-directed T cells in MPM patients.

